# Identification and validation of P4HB as a novel autophagy-related biomarker in diabetic nephropathy

**DOI:** 10.3389/fgene.2022.965816

**Published:** 2022-09-26

**Authors:** Fang Bai, Kuipeng Yu, Yanjiang Yang, Yimeng Zhang, Lin Ding, Xin An, Feng Feng, Nan Sun, Jiahui Fan, Lei Liu, Huimin Yang, Xiangdong Yang

**Affiliations:** ^1^ Department of Nephrology, Qilu Hospital of Shandong University, Jinan, Shandong, China; ^2^ Laboratory of Basic Medical Sciences, Qilu Hospital of Shandong University, Jinan, Shandong, China; ^3^ Department of General Practice, Qilu Hospital of Shandong University, Jinan, Shandong, China

**Keywords:** autophagy, P4HB, bioinformatics analysis, diabetic nephropathy, biomarker

## Abstract

Diabetic nephropathy (DN), a frequent microvascular complication of diabetes, has been recognized as a primary cause of chronic kidney disease (CKD) and end-stage renal disease (ESRD). Previous studies found that autophagy of renal tubular epithelial cells plays an important role in DN pathogenesis. Our research aimed to investigate the differentially expressed autophagy-related genes (DEARGs) between DN and healthy renal tubule samples and identify a novel autophagy-related biomarker associated with tubulointerstitial injury in DN. In this study, gene expression profiles of renal tubules from 10 DN patients and 24 healthy controls in the GSE30122 dataset were analyzed, and 43 DEARGs were identified by bioinformatics analysis. The Gene Ontology (GO) and Kyoto Encyclopedia of Genes and Genomes (KEGG) enrichment analysis and correlation analysis were performed on DEARGs, and the hub gene prolyl 4-hydroxylase subunit beta (*P4HB*) was screened by protein–protein interaction and verified by utilizing other datasets and stimulating HK-2 cells under high glucose concentration. We found that the expression of *P4HB* in renal tubules was correlated with renal function. In summary, our research provided novel insights for comprehension of DN molecular mechanisms and identified *P4HB* as a novel autophagy-related biomarker of DN.

## Introduction

Diabetic nephropathy (DN), a common microvascular complication of diabetes, has been recognized as a primary cause of chronic kidney disease (CKD) and end-stage renal disease (ESRD) in many developed and developing countries (Bikbov et al., 2020; Johansen et al., 2021). According to recent reports, DN has accounted for 20 %–40 % of patients requiring kidney replacement therapy worldwide, contributing to more than 950,000 deaths worldwide each year (Tang and Yiu, 2020). DN has become a serious global healthcare problem.

Although glomerular injury is a major pathological manifestation of DN, there is growing evidence suggesting that tubulointerstitial pathological changes, such as tubular atrophy, interstitial fibrosis, autophagy, and apoptosis of tubular epithelial cells, play a crucial role in DN development (Brezniceanu et al., 2010; C. Yang et al., 2021a; Liu et al., 2022). Apart from all other reasons, non-enzymatic glycation is considered one of the major reasons behind diabetes, including DN. It was found that DNA-AGEs and auto-antibodies against glycated DNA are related to diabetic microvascular complications, such as DN (Ahmad et al., 2014). At the same time, it was also reported that some commonly used drugs like ezetimibe and rosuvastatin can show strong antidiabetic and renal protective effects by targeting AGE/RAGE-associated signaling (Nabi et al., 2021a; Nabi et al., 2021b). In addition, some common components in plants such as ellagic acid and lycopene play a protective role in diabetes and DN by reducing the formation of AGEs (Tabrez et al., 2015; Ahmad et al., 2022).

Autophagy is a cellular process in which damaged organelles, protein aggregates, and other macromolecules are degraded in the cytoplasm (Galluzzi et al., 2017). Autophagy dysfunction is associated with pathogenesis of various diseases (Dikic and Elazar, 2018; C. Zhang et al., 2020). Several pathways affect the biological function of DN by influencing autophagy. Li et al. (2021) reported that activation of the epidermal growth factor receptor (EGFR) signaling pathway can exacerbate kidney damage by inhibiting autophagy. However, autophagy-related genes (ARGs) in DN are still largely unknown and require further exploration. Exploration and illumination of differentially expressed autophagy-related genes (DEARGs) in DN will provide us with novel biomarkers for treatment of DN. Bioinformatics is a method for efficiently and accurately processing large quantities of data, providing valuable information to patients. Nevertheless, studies on the investigation of the expression of diabetic renal tubular interstitial genes and autophagy through bioinformatics are still lacking.

**FIGURE 1:**
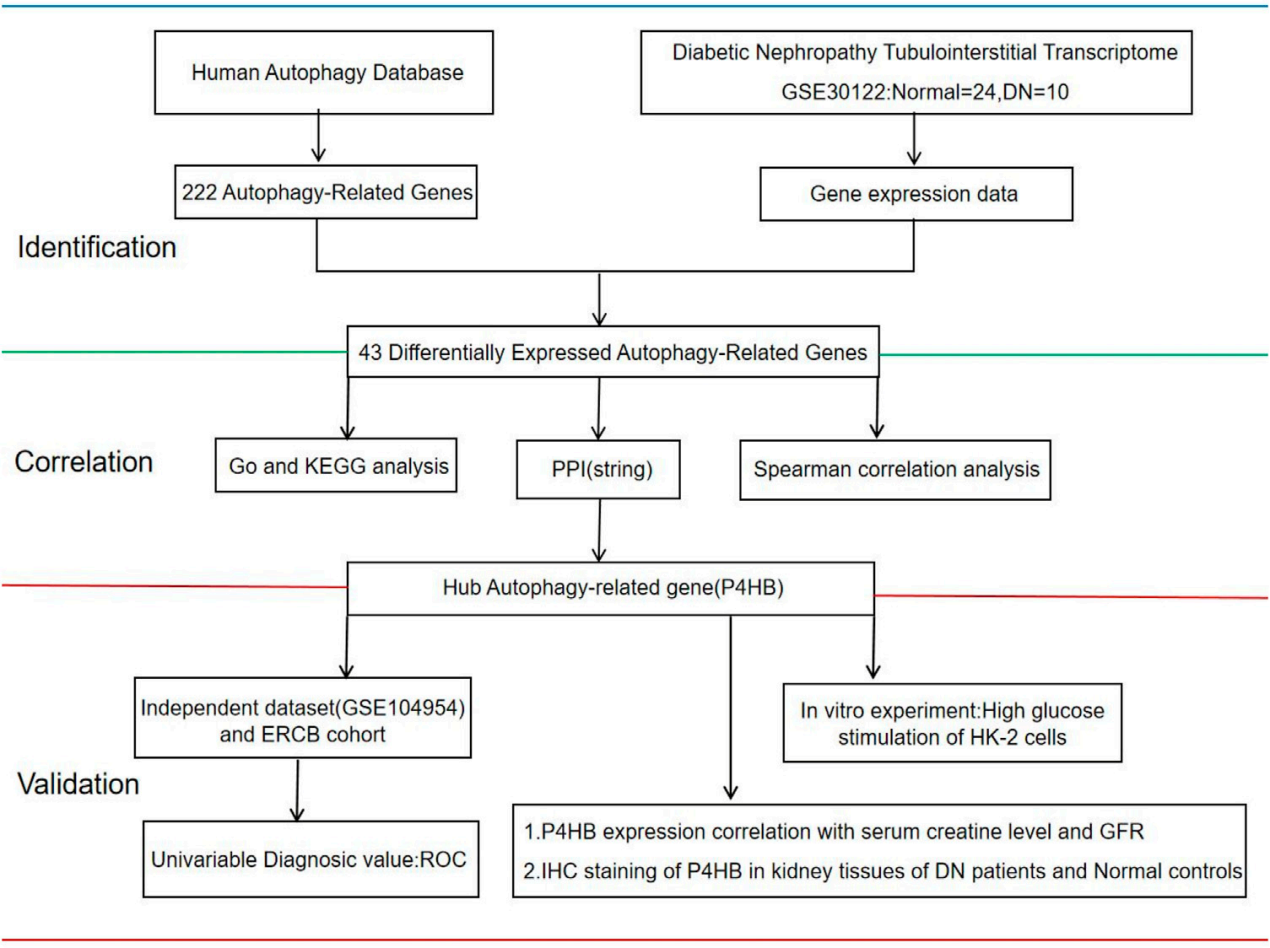
Workflow of this study. The gene expression of human renal tubulointerstitial data was extracted from the GEO database (GSE30122). GEO, Gene Expression Omnibus; Normal, healthy controls; DN, patients with diabetic nephropathy; GO, Gene Ontology; KEGG, Kyoto Encyclopedia of Genes and Genomes; PPI, protein–protein Interaction; ERCB: European Renal cDNA Bank; ROC, receiver operating curve; GFR, glomerular filtration rate; IHC, immunohistochemistry.

Woroniecka et al. completed the DN-related dataset GSE30122 for analysis of gene expression differences between DN patients and healthy controls (Woroniecka et al., 2011). In this study, we re-analyzed their dataset from other perspectives to explore DEARGs in DN vs. normal human renal tubular interstitial genes. Subsequently, we performed Gene Ontology (GO) enrichment analysis, Kyoto Encyclopedia of Genes and Genomes (KEGG) pathway enrichment analysis, and correlation analysis on DEARGs. Protein–protein interaction (PPI) showed that prolyl 4-hydroxylase subunit beta (*P4HB*) was identified as an autophagy-related hub gene for DN. Finally, we further validated the upregulated expression of *P4HB* in DN tubulointerstitium by exploiting another database (GSE104954) (Grayson et al., 2018) and the European Renal cDNA Bank (ERCB) cohort, as well as establishing an *in vitro* model, and the expression of *P4HB* in renal tubules was correlated with renal function. The experiments’ schematic workflow is displayed in Figure 1. Our study suggested that *P4HB* is a potential key biomarker in the pathogenesis of renal tubular injury in DN.

## Materials and methods

### Data download and preprocessing

The Human Autophagy Database (HADb; http://www.autophagy.lu/index.html) was used to obtain genes involved in autophagy. Genomic and transcriptomic datasets of DN and healthy renal tubule samples were obtained using Gene Expression Omnibus (GEO) (http://www.ncbi.nlm.nih.gov/geo/). Data from GSE30122 (GPL571 platform, Affymetrix Human Genome U133A 2.0 Array) included the data of 10 patients with DN (GSM757014–GSM757023) and 24 control human kidney tubules (GSM757024–GSM757035, GSM758498–GSM758509). The basic characteristics of 10 DN patients are summarized in Supplementary Table S1, and the histological evaluation of the 10 DN patients showed that renal tubular atrophy, interstitial fibrosis, vascular sclerosis, and mesangial matrix dilatation increased significantly (Woroniecka et al., 2011). Data from GSE104954 (GPL22, 945 platform, Affymetrix Human Genome U133 Plus 2.0 Array, and GPL 24,120 platform, Affymetrix Human Genome U133A Array) were used for validation. The “sva” (Parker et al., 2014) and “limma” (Ritchie et al., 2015) packages in R software were used to normalize raw data in batches.

### Differentially expressed autophagy-related gene analysis

The reproducibility of the GSE30122 data was examined using the principal component analysis (PCA) method. The “limma” package was utilized to investigate the differential expression of genes related to autophagy. Genes were identified as differentially expressed genes based on an adjusted *p*-value of <0.05 and an absolute fold-change value of >1.5. Heatmaps were created using the “pheatmap” package in R, and the volcano plots and box plots were performed using the Sangerbox tools, a free online platform for data analysis (http://vip.sangerbox.com/home.html).

### Gene functions and correlation analysis

Enrichment analyses were conducted for DEARGs. GO and KEGG pathway enrichment analyses were performed using DAVID version 6.8 (https://david.ncifcrf.gov/conversion.jsp), a commonly used tool for detailed analysis and classification of genes and protein functions in bioinformatics research. The GO analysis included cellular composition (CC), biological process (BP), and molecular function (MF). Enrichment results and correlation analysis were drawn by https://www.bioinformatics.com.cn, a free online platform for data analysis and visualization.

### Protein–protein interaction network construction

The PPI network was constructed on the basis of the Search Tool for the Retrieval of Interacting Genes/Proteins (STRING) online analysis (https://string-db.org/). PPI network visualization and analyses were performed with Cytoscape (Version 3.9.1). Thirty hub genes related to autophagy were identified by the Density of Maximum Neighborhood Component (DMNC), and *P4HB* was screened as a hub gene. An online platform (https://www.bioinformatics.com.cn) was also used to show the receiver operating curve (ROC) to evaluate the ability of *P4HB* to discriminate between DN patients from healthy controls.

### *P4HB* validation and correlation analysis with renal function

The differential gene expression of *P4HB* between DN and healthy renal tubule samples was verified in the GSE104954 dataset and ERCB cohort (31 healthy controls and 17 DN patients) (Ju et al., 2015). Then, the association analysis of *P4HB* expression and clinical characteristics was validated in ERCB using Pearson’s correlation analysis by using the Nephroseq v5 online database (http://v5.nephroseq.org).

### Cell culture and treatments

The human proximal tubular cell line HK-2 was purchased from the National Collection of Authenticated Cell Cultures and cultured in Dulbecco’s modified Eagle’s medium (c11885500BT, Gibco) by adding 10 % fetal bovine serum (10270-106, Gibco) and 1 % penicillin–streptomycin (p1400, Solarbio) at 37°C and 5 % CO_2_ in a humidified environment. High glucose concentration (30 mM, G8644, Sigma) for 24 h (Zhan et al., 2015) was used to cause HK-2 cell damage, and the addition of 5.5 mmol/L glucose served as the control.

### RNA extraction and RT-qPCR analysis

Total RNA was extracted from cultured HK-2 cells using the RNAfast200 Kit (Fastagen), and cDNA was synthesized using the SureScriptTM First-Stand cDNA Synthesis Kit (QP056T, GeneCopoeia). Real-time quantitative polymerase chain reaction (RT-qPCR) was conducted using SYBR Green reagent (1725201, Bio-Rad) on a Bio-Rad CFX PCR System (Bio-Rad). The procedure was repeated three times for each sample. The primers are given in Table 1. Gene expression analysis was performed using the 2^−ΔΔCt^ method, and expression levels were normalized to those of *GAPDH*.

**TABLE 1:** Quantitative PCR primers used in the study.

Gene	Primer sequence (from 5′ to 3′)
*P4HB*	Forward: 5′-CTG​CGG​AAA​AGC​AAC​TTC​GC-3′
	Reverse: 5′-CCA​CAC​CAA​GGG​GCA​TAG​AA-3′
*NGAL*	Forward: 5′-AGC​ACC​AAC​TAC​AAC​CAG​CAT-3′
	Reverse: 5′-TTG​GGA​CAG​GGA​AGA​CGA​TG-3′
*GAPDH*	Forward: 5′-GCA​CCG​TCA​AGG​CTG​AGA​AC-3′
	Reverse: 5′-TGG​TGA​AGA​CGC​CAG​TGG​A-3′

### Protein extraction and Western blotting analysis

Total proteins were extracted by the incubation of cultured HK-2 cells with radioimmunoprecipitation assay (RIPA) buffer (P0013D, Beyotime) and adding 1 % phenylmethylsulfonyl fluoride (PMSF) (329-98-6, Solarbio) and quantified by Bio-RAD assays. The same amount of protein was separated by 10 % sodium dodecyl sulfate (SDS)-polyacrylamide gel electrophoresis (SDS-PAGE), and proteins were transferred to polyvinylidene difluoride (PVDF) membranes. The membranes were then blocked with 5 % skim milk and subsequently incubated with primary antibodies against *P4HB* (1:1,000 dilution, Cat.137,110, Abcam) and GAPDH (1:6,000 dilution, Cat. 60004-1-Ig, ProteinTech) overnight at 4°C. After washing, the proteins were incubated with horseradish peroxidase (HRP)-conjugated secondary antibody (1:6,000 dilution, Cat. SA00001-2, ProteinTech) for 1 h at room temperature. The bands were visualized using an enhanced chemiluminescence (ECL) system, and the signal intensity was quantitatively processed by ImageJ software.

### Immunohistochemistry and immunofluorescent staining

The paraffin-embedded kidney sections of the kidney tissues of three DN patients diagnosed by renal biopsy from the pathology department of Qilu Hospital affiliated with Shandong University and the paraffin-embedded kidney sections of healthy adjacent kidney tissues of three individuals who underwent tumor nephrectomy (no diabetes or other kidney diseases) were immunohistochemically stained by *P4HB*, as approved by the Research Ethics Committee Qilu Hospital of Shandong University (NO. KYLL-2020(KS)-030). These sections were incubated with an anti-P4HB antibody (1:1,000 dilution, Cat.137110, Abcam) at 4°C overnight. The general two-step method was used for detection and incubation, and the 3, 3′-diaminobenzidine (DAB) chromogenic kit was used for immunohistochemical staining. Images were collected and analyzed using the NIS Element software and Nikon microscope imaging system, and ImageJ software was used for quantitative analysis.

For immunofluorescent staining of cells, the cells were fixed with 4 % paraformaldehyde, incubated with TritonX-100 (1 %) for 20 min, and then incubated with the *P4HB* primary antibody (1:200 dilution, Cat.137110, Abcam) overnight at 4°C, Then, the cells were incubated with goat anti-rabbit immunoglobulin (Ig)G DyLight 594 (1:500, Cat. A23420, Abbkine Scientific Company) coupled with a fluorescent probe in the dark at room temperature for 1 h, and the nuclei were observed by DAPI (AR1176, Boster Bio) staining. Images were captured by a fluorescence microscope (Olympus).

### Statistical analysis

GraphPad Prism 7 and R software version 4.1.3 were used for statistical analysis. A two-tailed unpaired *t* test was used for comparisons between the two groups. Differences were considered statistically significant at *p* <0.05. (*p < 0.05, **p < 0.01, ***p < 0.001, and ****p < 0.0001).

## Results

### Identification of differentially expressed autophagy-related genes between the DN and healthy tubule samples

**FIGURE 2:**
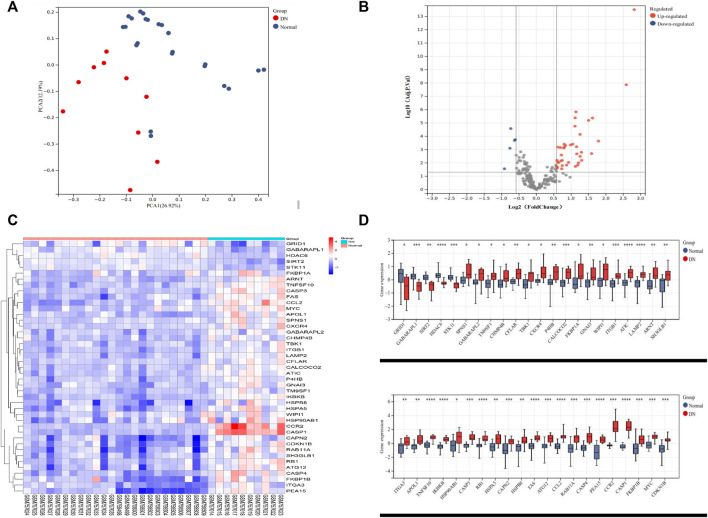
Differentially expressed autophagy-related genes in the tubulointerstitium between DN and normal renal tissue. **(A)** PCA for GSE30122. **(B)** Volcano plot of 222 DEARGs in DN and normal samples. **(C)** Heatmap of the 43 DEARGs in DN and normal samples. **(D)** Boxplot of the 43 DEARGs in DN and normal samples. *p < 0.05, **p < 0.01, ***p < 0.001, and ****p < 0.0001. PCA, principal component analysis; DEARG, differentially expressed autophagy-related gene; DN, diabetic nephropathy.

The renal tubular transcriptome data GSE10322 were used for further analysis to investigate the role of ARGs in DN pathogenesis. The distribution of DN and normal samples was shown by the PCA results (Figure 2A). The expression of 222 ARGs in 10 DN patients and 24 normal samples was subsequently analyzed, and 43 ARGs showed differential expression in DN with an adjusted *p* value <0.05 and an absolute fold-change value of >1.5, including 38 upregulated and 5 downregulated genes (Table 2; Figures 2B–D).

**TABLE 2:** 43 differentially expressed autophagy-related genes in the tubulointerstitium between DN and normal renal tissue.

Gene symbol	logFC	Change	*p*-value	Adj.*p*-value	Chromosome
*CASP1*	2.815552955	Up	1.56195E-16	3.09266E-14	11q23
*CCR2*	2.58694031	Up	1.36828E-10	1.35459E-08	3p21.31
*PEA15*	1.786883586	Up	1.39952E-05	0.00023092	1q21.1
*CASP4*	1.615423503	Up	1.0762E-07	4.26175E-06	11q22.2-q22.3
*RAB11A*	1.5878937	Up	0.000249019	0.001972229	15q22.31
*CCL2*	1.499910231	Up	1.94226E-07	6.40947E-06	17q11.2-q12
*FKBP1B*	1.303245	Up	1.08E-03	6.40E-03	2p23.3
*CDKN1B*	1.297208099	Up	0.000191967	0.001583727	12p13.1-p12
*ATG12*	1.258743997	Up	0.000488666	0.003225195	5q21-q22
*FAS*	1.258208019	Up	3.27484E-06	7.20464E-05	10q24.1
*HSPB8*	1.211358555	Up	0.002146235	0.009656732	12q24.23
*CAPN2*	1.191207762	Up	0.003702217	0.014511648	1q41-q42
*HSPA5*	1.16304934	Up	0.000273748	0.002084694	9q33.3
*RB1*	1.155716399	Up	5.24217E-05	0.000610559	13q14.2
*CASP3*	1.138499883	Up	2.22484E-08	1.46839E-06	4q34
*HSP90AB1*	1.122569062	Up	0.005094094	0.01833874	6p12
*IKBKB*	1.117062221	Up	8.29611E-08	4.10657E-06	8p11.2
*TNFSF10*	1.110782311	Up	6.15161E-07	1.74003E-05	3q26
*MYC*	1.04536829	Up	2.70E-05	3.89E-04	8q24.21
*APOL1*	0.982670415	Up	3.40551E-05	0.000449528	22q13.1
*ITGA3*	0.926426396	Up	0.001907668	0.009212639	17q21.33
*SH3GLB1*	0.871043634	Up	0.003591573	0.014511648	1p22
*ARNT*	0.835171835	Up	3.97754E-05	0.00049222	1q21
*LAMP2*	0.806156698	Up	6.16193E-05	0.000658743	Xq24
*ATIC*	0.800030145	Up	2.75244E-05	0.000389274	2q35
*ITGB1*	0.788846065	Up	7.99052E-05	0.000753392	10p11.2
*WIPI1*	0.744366027	Up	0.004194894	0.015972865	17q24.2
*GNAI3*	0.74200647	Up	0.001143994	0.006471739	1p13
*FKBP1A*	0.7292738	Up	0.009201632	0.027604895	20p13
*CALCOCO2*	0.719431492	Up	0.000347926	0.002551457	17q21.32
*P4HB*	0.71230372	Up	0.00133895	0.006976636	17q25
*CXCR4*	0.692229997	Up	7.17148E-05	0.000709977	2q21
*TBK1*	0.641395881	Up	0.001994129	0.009400893	12q14.1
*CFLAR*	0.618087278	Up	6.32127E-05	0.000658743	2q33-q34
*TM9SF1*	0.614734304	Up	0.001742586	0.008625801	14q11.2
*CHMP4B*	0.60073086	Up	0.005999651	0.020736493	14q12
*GABARAPL2*	0.598015027	Up	0.007554347	0.024929345	16q22.1
*SPNS1*	0.592296801	Up	0.001098847	0.006399169	16p11.2
*STK11*	−0.607962624	Down	8.98484E-06	0.0001779	19p13.3
*HDAC6*	−0.628923381	Down	1.13142E-05	0.000203656	Xp11.23
*SIRT2*	−0.737375167	Down	1.05727E-06	2.61673E-05	19q13
*GABARAPL1*	−0.760812687	Down	8.81349E-05	0.000793214	15q26.1
*GRID1*	−0.919198606	Down	0.008745839	0.027486922	10q23.3

### Functional enrichment analysis of the differentially expressed autophagy-related genes

**FIGURE 3:**
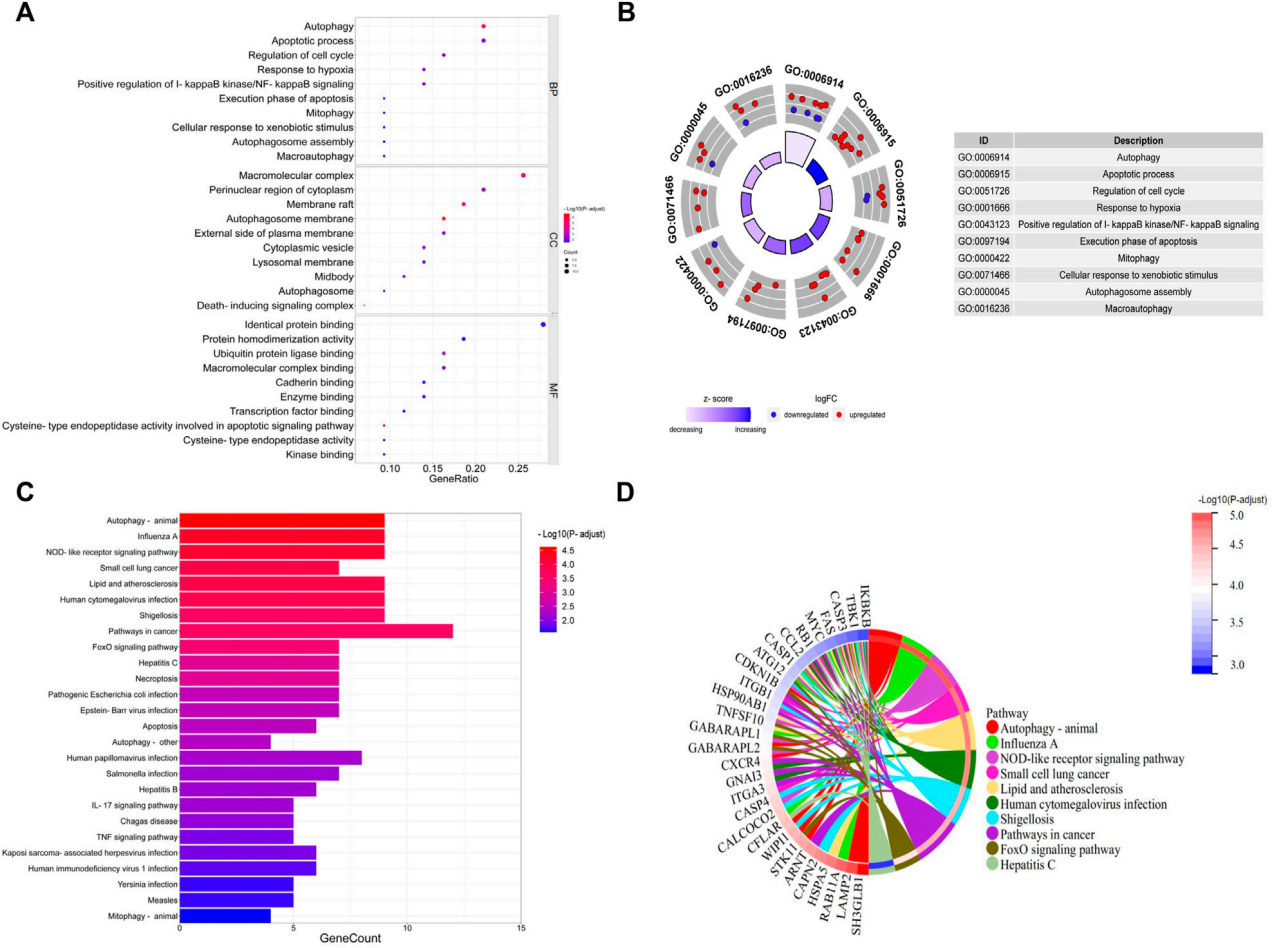
GO and KEGG enrichment analyses of 43 differentially expressed autophagy-related genes. **(A, B)** Bubble plot and circle plot of GO enrichment terms. BP, biological process; CC, cellular component; MF, molecular function. **(C, D)** Bar plot and Chord plot of KEGG enrichment pathways. GO, Gene Ontology; KEGG, Kyoto Encyclopedia of Genes and Genomes.

GO and KEGG pathway enrichment analyses were performed to further explore the biological functions of these DEARGs. The most involved processes or components in the GO included autophagy and apoptotic process (biological process), autophagosome membrane and membrane raft (cell component), and cysteine-type endopeptidase activity involved in the apoptotic signaling pathway and ubiquitin protein ligase binding (molecular function) (Figures 3A, B; Supplementary Table S2). The DEARGs mainly involved in the autophagy-associated process were shown by the KEGG pathway enrichment analysis (Figures 3C, D; Supplementary Table S3).

### Hub gene identification and validation

**FIGURE 4:**
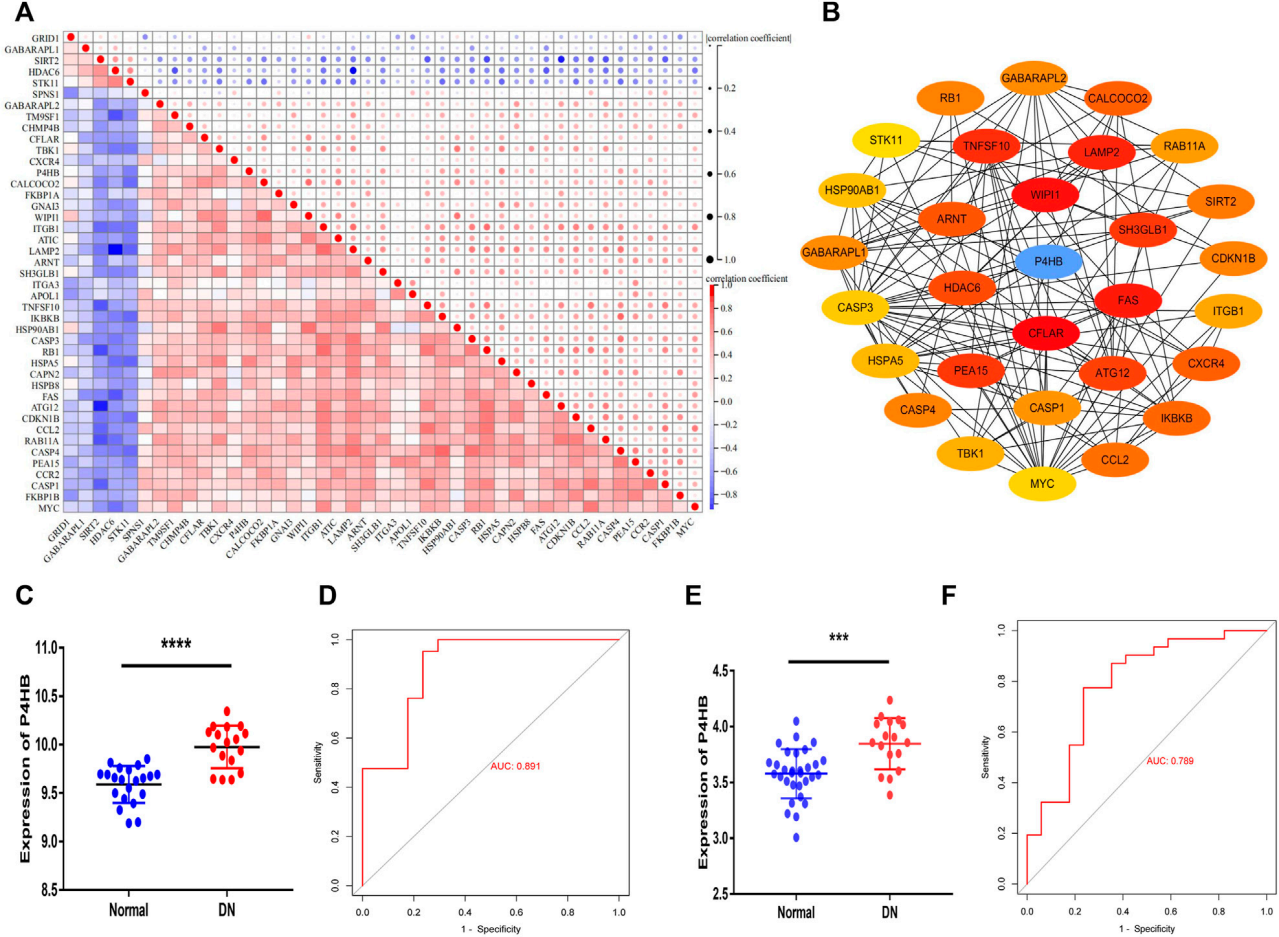
Identifying *P4HB* as a hub autophagy-related gene in DN and validating in the GSE104954 database and ERCB cohort. **(A)** Spearman’s correlation analysis of the 43 DEARGs. **(B)** Top 30 hub DEARGs identified *via* the PPI network. **(C, D)** Validation of *P4HB* in GSE104954. **(C)** Significantly upregulated expression of *P4HB* in DN patients (n = 17) compared to healthy samples (n = 21) (*p* < 0.0001) **(D)** ROC curve of *P4HB* expression in DN (AUC = 89.1%). **(E, F)** Validation of *P4HB* in the ERCB cohort. **(E)** Significantly upregulated expression of *P4HB* in DN patients (n = 17) compared to healthy samples (n = 31) (*p* < 0.0001). **(F)** ROC curve of *P4HB* expression in DN (AUC = 78.9%). *p < 0.05, **p < 0.01, ***p < 0.001, and ****p < 0.0001. DEARG, differentially expressed autophagy-related gene; DN, diabetic nephropathy; PPI, protein–protein Interaction; ROC, receiver operating curve; AUC, area under the curve; ERCB: European Renal cDNA Bank.

A correlation analysis was conducted to investigate the expression relevance of these DEARGs. The relationship of 43 DEARGs in the GSE30122 dataset is shown in Figure 4A. The human protein interaction database (String) was chosen to identify the interactions among DEARGs, and 30 hub genes were calculated by the DMNC algorithm. *P4HB* was screened as a hub DEARG in DN (Figure 4B). We utilized another independent dataset GSE104954 and an ERCB cohort to further verify the change in *P4HB* expression, showing that *P4HB* expression was significantly upregulated in DN tubule samples (Figures 4C–F). Additionally, the ROC analysis demonstrated that the expression of P4HB showed an excellent diagnostic value for DN patients and healthy controls (Figures 4C–F **)**.

### Validation of *P4HB* expression under high glucose stimulation

**FIGURE 5:**
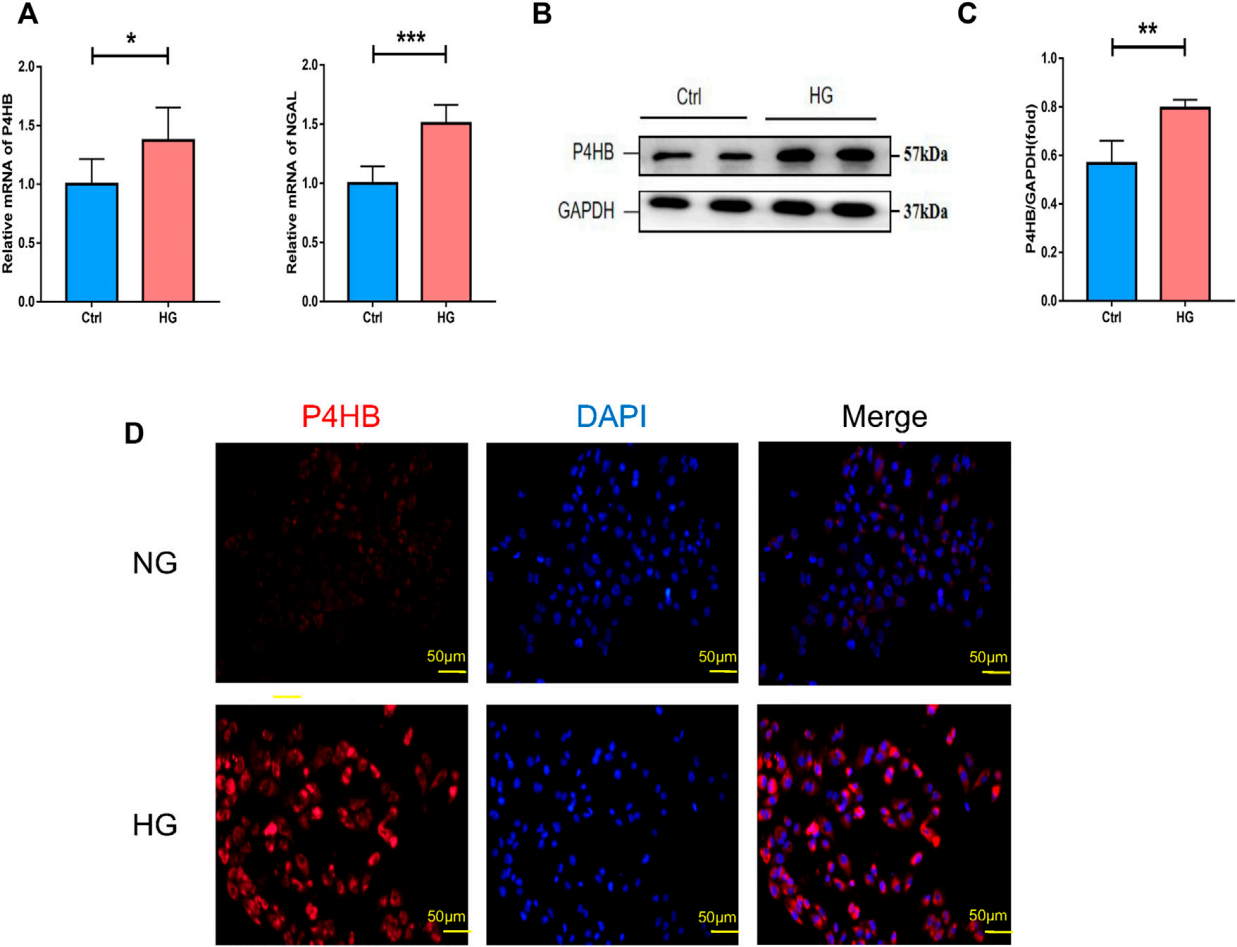
Validation of *P4HB* expression *in vitro*. **(A)** RT-qPCR analysis of *P4HB* and *NGAL* in HK-2 cells after HG stimulation and NG treatment. **(B)** Western blot analysis of *P4HB* in HK-2 cells after HG stimulation and NG treatment. **(C)** Densitometric quantification of *P4HB* in HK-2 cells after HG stimulation and NG treatment. Results are expressed as the mean ± SEM. *p < 0.05, **p < 0.01, ***p < 0.001, and ****p < 0.0001. **(D)** Immunofluorescence showing the expression of *P4HB* in HK-2 after HG stimulation and NG treatment. Scale bars, 50 μm. RT-qPCR, real-time quantitative polymerase chain reaction; SEM, standard error of the mean; HG, high glucose; NG, normal glucose.

After determining from other datasets that the expression of *P4HB* was significantly elevated in DN patients, the transcriptional and protein levels of *P4HB* were explored after high glucose stimulation and normal glucose treatment of HK-2 cells. RT-qPCR (Figure 5A) and Western blot analysis (Figures 5B, C) further validated that *P4HB* expression was elevated in HK-2 cells under high glucose (30 mM) stimulation, accompanied by increased expression of NGAL, a marker for renal tubular injury, indicating that there was significant damage in HK-2 cells. Simultaneously, cell immunofluorescence showed that the expression of *P4HB* in HK-2 cells increased significantly after high glucose stimulation (Figure 5D).

### Clinical relevance of *P4HB* expression and immunohistochemical validation of *P4HB* expression

**FIGURE 6:**
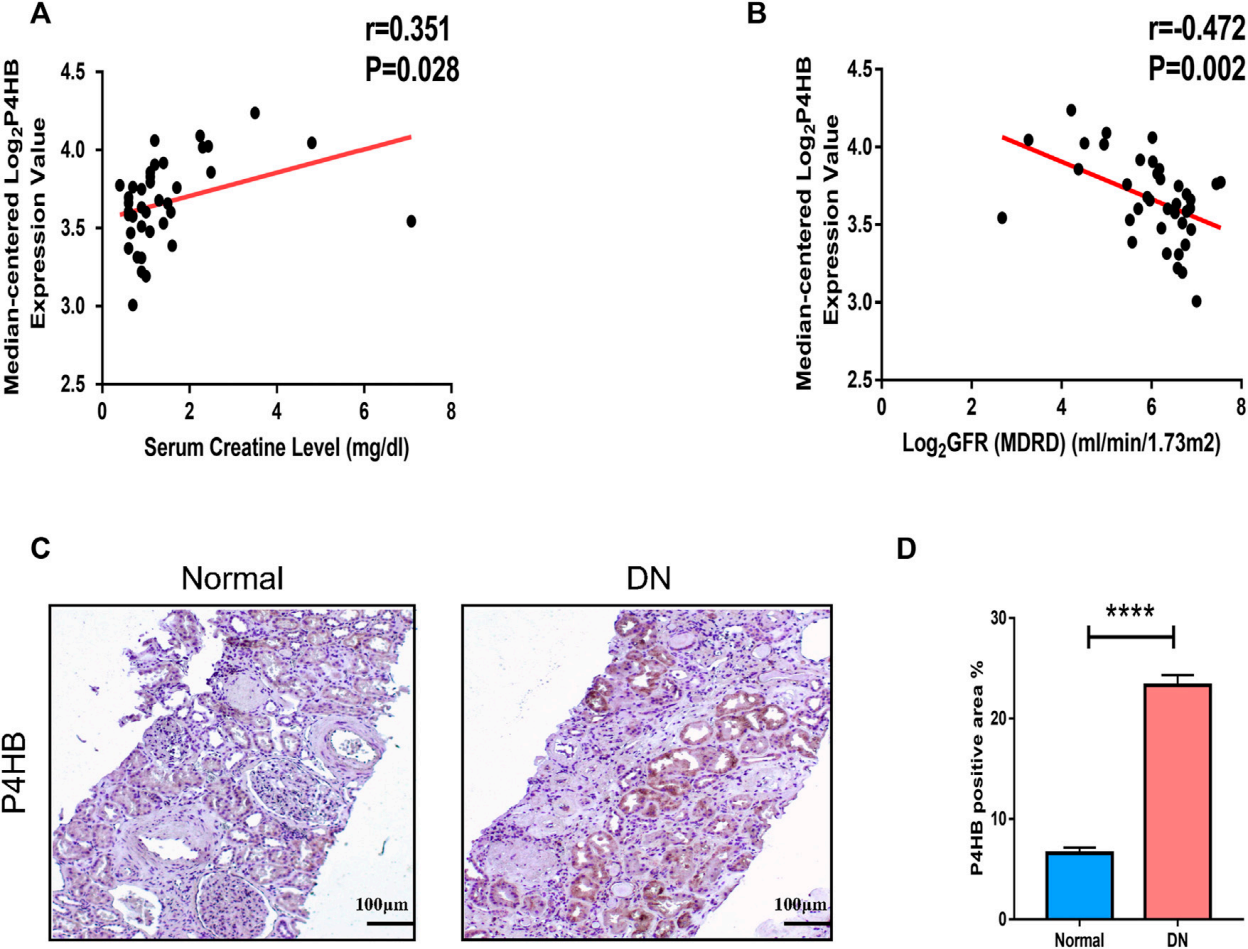
Clinical relevance of *P4HB* and immunohistochemical validation of P4HB expression. **(A)** Relevance of the expression of *P4HB* and serum creatinine level. **(B)** Relevance of the expression of *P4HB* and GFR. **(C,D)** Typical images and statistical charts of immunohistochemical staining of *P4HB* in normal renal tissues and DN renal tissues. Scale bars, 100 μm. Results are expressed as the mean ± SEM. *p < 0.05; **p < 0.01; ***p < 0.001, and ****p < 0.0001. GFR, glomerular filtration rate; DN, diabetic nephropathy; SEM, standard error of the mean.

The correlation analysis of the ERCB cohort was performed to validate the relationship between the expression of *P4HB* in renal tubular and renal function. *P4HB* expression was positively correlated with serum creatinine levels (r = 0.351, *p* = 0.028) and negatively correlated with glomerular filtration rate (GFR) (r = −0.472, *p* = 0.002) (Figures 6A,B). Additionally, we performed immunohistochemical staining of *P4HB* in kidney tissues of healthy controls and DN patients, and found that the expression of *P4HB* in the renal tubules of DN patients was significantly higher than that in normal kidneys (Figures 6C,D **)**.

## Discussion

DN is one of the most serious microvascular complications of diabetes, accounting for approximately 30 %–40 % of end-stage kidney disease patients worldwide (Maezawa, Takemoto and Yokote., 2015; L. Zhang et al., 2016). Poor prognosis and low quality of life are distinctive characteristics of patients with DN (Slieker et al., 2021). Therefore, novel and satisfactory strategies are urgently needed to treat DN. There is growing evidence that multiple biological functions are engaged in the pathogenesis of DN, such as immunity, inflammation, and autophagy (Yang et al., 2019; Yang M. et al., 2021; Yu et al., 2021; Li et al., 2022). In our research, starting from autophagy, for the first time, we identified DEARGs in DN vs. normal human renal tubular interstitial cells by bioinformatics analysis, as well as determined a new autophagy-related biomarker for DN, thus providing novel insights into the tubulointerstitial pathogenesis of DN and contributing to the identification of novel therapeutic targets.

*P4HB*, a member of the protein disulfide isomerase (PDI) family, is a multifunctional protein capable of catalyzing the generation and reorganization of disulfide bonds (Noiva, 1999). *P4HB*, as an autophagy-related gene, can be detected in various diseases that involve inflammation and apoptosis, including cancer, endocrine diseases, and skin diseases. Elevated expression of *P4HB* has been reported in several solid tumors, such as ovarian cancer (Bonome et al., 2008), bladder cancer (Lyu et al., 2020; Wang et al., 2020), and prostate cancer (Welsh et al., 2001; Singh et al., 2002). Additionally, Ding et al. (2020) reported that targeting *P4HB* can reduce inflammation and melanogenesis of the skin. Furthermore, previous studies found that PDIA1 contributes to oxidative maturation of proinsulin in the endoplasmic reticulum to support insulin production and ß-cell health in diet-induced obesity (Jang et al., 2019), indicating that *P4HB* could indirectly influence insulin production and ß-cell health.

One study has determined that *P4HB* was substantially elevated as an ARG in kidney renal clear cell carcinoma (KIRC) and showed high diagnostic and prognostic ability (Xie et al., 2020). There are also reports demonstrating that *P4HB* overexpression was associated with poor prognosis in human KIRC (Zhu et al., 2019; Wu et al., 2021), indicating that overexpression of *P4HB* is an adverse prognostic factor. Additionally, Fu et al. (2021) reported that the self-antigen *P4HB* located on the cell membrane of kidney cells could be cross-recognized by anti-HU1 (a conserved peptide derived from DNABII proteins) and induce lupus nephritis (LN). Hence, evidence suggests that *P4HB* might be a key biomarker and therapeutic target for human kidney diseases.

In this study, transcriptomic variations of ARGs in the gene expression profiles of 10 DN and 24 healthy renal tubule samples were analyzed, and 43 DEARGs were identified in DN samples compared with healthy samples. The GO and KEGG enrichment analyses of DEARGs were subsequently performed. These genes were mostly enriched in autophagy-related biological processes, such as autophagy, mitophagy, autophagosome assembly, and macroautophagy. These processes may be associated with various infections and diseases that were inferred from the enrichment analysis of pathways, including influenza A, lipid dysfunction and atherosclerosis, and cancer. Previous studies have also shown that DN was associated with various autophagy-related biological functions (Yang M. et al., 2021; Liu et al., 2022). Then, correlation analysis and the PPI network were constructed to further explore the correlation between the DEARG expression. According to the PPI network, DMNC scores from cytoHubba confirmed that *P4HB* was a key hub gene in DN tubular injury.

The elevation of renal tubular *P4HB* expression in DN was verified in other datasets and *in vitro* experiments. We first verified the elevated gene expression by other databases, and the result was consistent with that of our previous study. The ROC analysis indicated that *P4HB* expression showed an excellent diagnostic value for DN patients and healthy controls. Through *in vitro* experiments, we found that the expression of *P4HB* was significantly elevated after high glucose stimulation of HK-2, accompanied by increased expression of NGAL, a marker for kidney tubular injury, indicating that there was significant damage in HK-2 cells (Satirapoj, 2018). In the meantime, we found that *P4HB* expression was positively correlated with serum creatinine levels and negatively correlated with GFR, and immunohistochemistry staining showed that the expression of P4HB in the renal tubules of DN patients was significantly higher than that of normal kidneys. Therefore, *P4HB* might serve as an autophagy-related biomarker for DN.

This study can provide novel insights and potential targets for further studies on the connection between DN and autophagy. However, our analysis was limited by the number of samples included, as transcriptomic data on DN tubuleinterstitium are restricted and most data were tested with different platforms. More combined samples and clinical information are required to clarify the potential mechanisms of *P4HB* in DN.

## Conclusion

We identified DEARGs in DN vs. normal human renal tubular interstitial cells by bioinformatics analysis for the first time, and *P4HB* was found and confirmed as an autophagy-related biomarker for DN, thereby providing new insights and potential targets for further studies on the correlation between DN and autophagy.

## Data Availability

The datasets presented in this study can be found in online repositories. The names of the repository/repositories and accession number(s) can be found below:, GSE30122; further inquiries can be directed to the corresponding author.

## References

[B1] Ahamd S. Alouffi S. Khan S. Khan M. Akasha R. Ashraf J. M. (2022). Physicochemical characterization of *in vitro* LDL glycation and its inhibition by ellagic acid (EA): An *in vivo* approach to inhibit diabetes in experimental animals. Biomed. Res. Int. 2022, 5583298. 10.1155/2022/5583298 35097119 PMC8791751

[B2] Ahamd S. MmoinuddinShahab U. Habisb S. Khan M. S. Alam K. Ali A. (2014). Glycoxidative damage to human DNA: Neo-antigenic epitopes on DNA molecule could be a possible reason for autoimmune response in type 1 diabetes. Glycobiology 24 (3), 281–291. 10.1093/glycob/cwt109 24347633

[B3] Bikbov B. Purcell C. Levey A. S. Smith M. Abdoli A. Abebe M. (2020). Global, regional, and national burden of chronic kidney disease, 1990-2017: A systematic analysis for the global burden of disease study 2017. Lancet 395 (10225), 709–733. 10.1016/s0140-6736(20)30045-3 32061315 PMC7049905

[B4] Bonome T. Levine D. A. Shih J. Randonovich M. Pise-Masison C. A. Bogomolniy F. (2008). A gene signature predicting for survival in suboptimally debulked patients with ovarian cancer. Cancer Res. 68 (13), 5478–5486. 10.1158/0008-5472.Can-07-6595 18593951 PMC7039050

[B5] Brezniceanu M.-L. Lau C. J. Godin N. Chenier I. Duclos A. Ethier J. (2010). Reactive oxygen species promote caspase-12 expression and tubular apoptosis in diabetic nephropathy. J. Am. Soc. Nephrol. 21 (6), 943–954. 10.1681/asn.2009030242 20299359 PMC2900966

[B6] Dikic I. Elazar Z. (2018). Mechanism and medical implications of mammalian autophagy. Nat. Rev. Mol. Cell Biol. 19 (6), 349–364. 10.1038/s41580-018-0003-4 29618831

[B7] Ding X.-J. Zhang Z.-Y. Jin J. Han J.-X. Wang Y. Yang K. (2020). Salidroside can target both P4HB-mediated inflammation and melanogenesis of the skin. Theranostics 10 (24), 11110–11126. 10.7150/thno.47413 33042273 PMC7532676

[B8] Fu W. Liu Y. Liu F. Liu C. Li J. Niu J. (2021). A novel autoantibody induced by bacterial biofilm conserved components aggravates Lupus nephritis. Front. Immunol. 12, 656090. 10.3389/fimmu.2021.656090 33841441 PMC8027312

[B9] Galluzzi L. Baehrecke E. H. Ballabio A. Boya P. Pedro J. M. B.-S. Cecconi F. (2017). Molecular definitions of autophagy and related processes. Embo J. 36 (13), 1811–1836. 10.15252/embj.201796697 28596378 PMC5494474

[B10] Grayson P. C. Eddy S. Taroni J. N. Lightfoot Y. L. Mariani L. Parikh H. (2018). Metabolic pathways and immunometabolism in rare kidney diseases. Ann. Rheum. Dis. 77 (8), 1226–1233. 10.1136/annrheumdis-2017-212935 29724730 PMC6045442

[B11] Jang I. Pottekat A. Poothong J. Yong J. Lagunas-Acosta J. Charbono A. (2019). PDIA1/P4HB is required for efficient proinsulin maturation and ß cell health in response to diet induced obesity. Elife 8, e44528. 10.7554/eLife.44528 31184304 PMC6559792

[B12] Johansen K. L. Chertow G. M. Foley R. N. Gilbertson D. T. Herzog C. A. Ishani A. (2021). US renal data system 2020 annual data report: Epidemiology of kidney disease in the United States. Am. J. Kidney Dis. 77, A7–A8. 10.1053/j.ajkd.2021.01.002 33752804 PMC8148988

[B13] Ju W. Nair V. Smith S. Zhu L. Shedden K. Song P. X. K. (2015). Tissue transcriptome-driven identification of epidermal growth factor as a chronic kidney disease biomarker. Sci. Transl. Med. 7 (316), 316ra193. 10.1126/scitranslmed.aac7071 PMC4861144 26631632

[B14] Li A. Yi B. Han H. Yang S. Hu Z. Zheng L. (2022). Vitamin D-VDR (vitamin D receptor) regulates defective autophagy in renal tubular epithelial cell in streptozotocin-induced diabetic mice via the AMPK pathway. Autophagy 18 (4), 877–890. 10.1080/15548627.2021.1962681 34432556 PMC9037529

[B15] Li Y. Pan Y. Caouasasaki S. K. Wang Y. Niu A. Fan X (2021). Podocyte EGFR inhibits autophagy through upregulation of rubicon in type 2 diabetic nephropathy. Diabetes 70 (2), 562–576. 10.2337/db20-0660 33239448 PMC7881855

[B16] Liu L. Bai F. Song H. Xiao R. Wang Y. Z. Yang H. M. (2022). Upregulation of TIPE1 in tubular epithelial cell aggravates diabetic nephropathy by disrupting PHB2 mediated mitophagy. Redox Biol. 50, 102260. 10.1016/j.redox.2022.102260 35152003 PMC8844679

[B17] Lyu L. Xiang W. Zheng F. Huang T. Feng Y. Yuan J. (2020). Significant prognostic value of the autophagy-related gene P4HB in bladder urothelial carcinoma. Front. Oncol. 10, 1613. 10.3389/fonc.2020.01613 32903592 PMC7438560

[B18] Maezawa Y. Takemoto M. Yokote K. (2015). Cell biology of diabetic nephropathy: Roles of endothelial cells, tubulointerstitial cells and podocytes. J. Diabetes Investig. 6 (1), 3–15. 10.1111/jdi.12255 PMC4296695 25621126

[B19] Nabi R. Alvi S. S. Alouffi S. Khan S. Ahmad A. Khan M. (2021a). Amelioration of neuropilin-1 and RAGE/matrix metalloproteinase-2 pathway-induced renal injury in diabetic rats by rosuvastatin. Arch. Biol. Sci. 73 (2), 265–278. 10.2298/abs210316021n

[B20] Nabi R. Alvi S. S. Shah A. Chaturvedi C. P. Faisal M. Alat A. A. (2021b). Ezetimibe attenuates experimental diabetes and renal pathologies via targeting the advanced glycation, oxidative stress and AGE-RAGE signalling in rats. Arch. Physiol. Biochem. 2021, 1–16. 10.1080/13813455.2021.1874996 33508970

[B21] Noiva R. (1999). Protein disulfide isomerase: The multifunctional redox chaperone of the endoplasmic reticulum. Semin. Cell Dev. Biol. 10 (5), 481–493. 10.1006/scdb.1999.0319 10597631

[B22] Parker H. S. Leek J. T. Favorov A. V. Considine M. Xia X. Chavan S. (2014). Preserving biological heterogeneity with a permuted surrogate variable analysis for genomics batch correction. Bioinformatics 30 (19), 2757–2763. 10.1093/bioinformatics/btu375 24907368 PMC4173013

[B23] Ritcheie M. E. Phipson B. Wu D. Hu Y. Law C. W. Shi W. (2015). Limma powers differential expression analyses for RNA-sequencing and microarray studies. Nucleic Acids Res. 43 (7), e47. 10.1093/nar/gkv007 25605792 PMC4402510

[B24] Satirapoj B. (2018). Tubulointerstitial biomarkers for diabetic nephropathy. J. Diabetes Res. 2018, 2852398. 10.1155/2018/2852398 29577044 PMC5822931

[B25] Singh D. Febbo P. G. Ross K. Jackson D. G. Manola J. Ladd C. (2002). Gene expression correlates of clinical prostate cancer behavior. Cancer Cell 1 (2), 203–209. 10.1016/s1535-6108(02)00030-2 12086878

[B26] Slieker R. C. Van Der Heijden A. A. W. A. Siddiqui M. K. Langendoen-Gort M. Nijpels G. Herings R. (2021). Performance of prediction models for nephropathy in people with type 2 diabetes: Systematic review and external validation study. Bmj-British Med. J. 374, n2134. 10.1136/bmj.n2134 PMC8477272 34583929

[B27] Tabrez S. Al-Shali K. Z. Ahmad S. (2015). Lycopene powers the inhibition of glycation-induced diabetic nephropathy: A novel approach to halt the AGE-RAGE axis menace. Biofactors 41 (5), 372–381. 10.1002/biof.1238 26453295

[B28] Tang S. C. W. Yiu W. H. (2020). Innate immunity in diabetic kidney disease. Nat. Rev. Nephrol. 16 (4), 206–222. 10.1038/s41581-019-0234-4 31942046

[B29] Wang X. Bai Y. Zhang F. Yang Y. Feng D. Li A. (2020). Targeted inhibition of P4HB promotes cell sensitivity to gemcitabine in urothelial carcinoma of the bladder. Onco. Targets. Ther. 13, 9543–9558. 10.2147/ott.S267734 33061438 PMC7532080

[B30] Welsh J. B. Sapinoso L. M. Su A. I. Kern S. G. Wang-rodriguez J. Moskaluk C. A. (2001). Analysis of gene expression identifies candidate markers and pharmacological targets in prostate cancer. Cancer Res. 61 (16), 5974–5978. 11507037

[B31] Woroniecka K. I. Park A. S. D. Mohtat D. Thomas D. B. Pullman J. M. Susztak K. (2011). Transcriptome analysis of human diabetic kidney disease. Diabetes 60 (9), 2354–2369. 10.2337/db10-1181 21752957 PMC3161334

[B32] Wu P. Xiang T. Wang J. Lv R. Ma S. Yuan L. (2021). Identification of immunization-related new prognostic biomarkers for papillary renal cell carcinoma by integrated bioinformatics analysis. BMC Med. Genomics 14 (1), 241. 10.1186/s12920-021-01092-w 34620162 PMC8499437

[B33] Xie L. Li H. Zhang L. Ma X. Dang Y. Guo J. (2020). Autophagy-related gene P4HB: A novel diagnosis and prognosis marker for kidney renal clear cell carcinoma. Aging 12 (2), 1828–1842. 10.18632/aging.102715 32003756 PMC7053637

[B34] Yang C. Chen X.-C. Li Z.-H. Wu H.-L. Jing K.-P. Huang X.-R. (2021a). SMAD3 promotes autophagy dysregulation by triggering lysosome depletion in tubular epithelial cells in diabetic nephropathy. Autophagy 17 (9), 2325–2344. 10.1080/15548627.2020.1824694 33043774 PMC8496726

[B35] Yang H. Xie T. Li D. Du X. Wang T. Li C. (2019). Tim-3 aggravates podocyte injury in diabetic nephropathy by promoting macrophage activation via the NF-kappa B/TNF-alpha pathway. Mol. Metab. 23, 24–36. 10.1016/j.molmet.2019.02.007 30862474 PMC6479760

[B36] Yang M. Luo S. Jiang N. Wang X. Han Y. Zhao H. (2021b). DsbA-L ameliorates renal injury through the AMPK/NLRP3 inflammasome signaling pathway in diabetic nephropathy. Front. Physiol. 12, 659751. 10.3389/fphys.2021.659751 33995126 PMC8120163

[B37] Yu K. Li D. Xu F. Guo H. Feng F. Ding Y. (2021). Ido1 as a new immune biomarker for diabetic nephropathy and its correlation with immune cell infiltration. Int. Immunopharmacol. 94, 107446. 10.1016/j.intimp.2021.107446 33581581

[B38] Zhan M. Usman I. M. Sun L. Kanwar Y. S. (2015). Disruption of renal tubular mitochondrial quality control by myo-inositol oxygenase in diabetic kidney disease. J. Am. Soc. Nephrol. 26 (6), 1304–1321. 10.1681/asn.2014050457 25270067 PMC4446875

[B39] Zhang C. Jiang J. Wang L. Zheng L. Xu J. Qi X. (2020). Identification of autophagy-associated biomarkers and corresponding regulatory factors in the progression of colorectal cancer. Front. Genet. 11, 245. 10.3389/fgene.2020.00245 32265986 PMC7100633

[B40] Zhang L. Long J. Jiang W. Shi Y. He X. Zhou Z. (2016). Trends in chronic kidney disease in China. N. Engl. J. Med. 375 (9), 905–906. 10.1056/NEJMc1602469 27579659

[B41] Zhu Z. He A. Lv T. Xu C. Lin L. Lin J. (2019). Overexpression of P4HB is correlated with poor prognosis in human clear cell renal cell carcinoma. Cancer Biomark. 26 (4), 431–439. 10.3233/cbm-190450 31640086 PMC12826406

