# Supplementary material for: Identification and validation of P4HB as a novel autophagy-related biomarker in diabetic nephropathy
Source: Front Genet. 2022 Sep 26;13:965816. doi: 10.3389/fgene.2022.965816 (PMC9548632; doi:10.3389/fgene.2022.965816)
Supplement: Supplementary file 1 [file Table1.docx]

Supplementary Material

**Supplementary Table 1.** Basic features of 10 patients with DN in GSE30122.

| **Sample Number** | **Sample Name** | **Age (Years)** | **GFR (MDRD) (ml/min/1.73m2)** | **Race/Ethnicity** | **Sex** | **Tissue Type** |
| --- | --- | --- | --- | --- | --- | --- |
| 1 | 120_DKD_tub | 84 | 32.44211196 | Black or African American | Female | Tubulointerstitium |
| 2 | 148_DKD_tub | 75 | 24.40272619 | Asian | Female | Tubulointerstitium |
| 3 | 162_DKD_tub | 54 | 11 | Hispanic | Female | Tubulointerstitium |
| 4 | 164_DKD_tub | 76 | 16 | Asian | Male | Tubulointerstitium |
| 5 | 168_DKD_tub | 73 | 37.95131892 | Black or African American | Female | Tubulointerstitium |
| 6 | 178_DKD_tub | 70 | 18 | Asian | Female | Tubulointerstitium |
| 7 | 187_DKD_tub | 47 | 18 | Black or African American | Female | Tubulointerstitium |
| 8 | 43_DKD_tub | 56 | 10 | Black or African American | Female | Tubulointerstitium |
| 9 | 62_DKD_tub | 33 | 10 | Black or African American | Female | Tubulointerstitium |
| 10 | 67_DKD_tub | 67 | 40.7832013 | Black or African American | Male | Tubulointerstitium |

**Supplementary Table 2.** Top 30 GO terms based on 43 differentially expressed autophagy-related genes in DN.

| **Ontology** | **ID** | **Description** | **Adj.P-value** | **Count** |
| --- | --- | --- | --- | --- |
| BP | GO:0006914 | Autophagy | 7.91E-07 | 9 |
| BP | GO:0097194 | Apoptotic process | 0.008272524 | 4 |
| BP | GO:0006915 | Regulation of cell cycle | 0.008272524 | 9 |
| BP | GO:0001666 | Response to hypoxia | 0.008272524 | 6 |
| BP | GO:0051726 | Positive regulation of I-kappaB kinase/NF-kappaB signaling | 0.008272524 | 7 |
| BP | GO:0043123 | Execution phase of apoptosis | 0.008272524 | 6 |
| BP | GO:0000422 | Mitophagy | 0.011856516 | 4 |
| BP | GO:0071466 | Cellular response to xenobiotic stimulus | 0.032352943 | 4 |
| BP | GO:0000045 | Autophagosome assembly | 0.032786867 | 4 |
| BP | GO:0016236 | Macroautophagy | 0.032786867 | 4 |
| CC | GO:0000421 | Autophagosome membrane | 1.88E-07 | 7 |
| CC | GO:0045121 | Membrane raft | 3.94E-05 | 8 |
| CC | GO:0032991 | macromolecular complex | 4.54E-05 | 11 |
| CC | GO:0031264 | Death-inducing signaling complex | 0.00219813 | 3 |
| CC | GO:0048471 | Perinuclear region of cytoplasm | 0.003111363 | 9 |
| CC | GO:0009897 | External side of plasma membrane | 0.006020552 | 7 |
| CC | GO:0005776 | Autophagosome | 0.008245549 | 4 |
| CC | GO:0031410 | Cytoplasmic vesicle | 0.008245549 | 6 |
| CC | GO:0030496 | Midbody | 0.008245549 | 5 |
| CC | GO:0005765 | Lysosomal membrane | 0.012870728 | 6 |
| MF | GO:0097199 | Cysteine-type endopeptidase activity involved in apoptotic signaling pathway | 2.24E-04 | 4 |
| MF | GO:0031625 | Ubiquitin protein ligase binding | 0.003120417 | 7 |
| MF | GO:0044877 | Macromolecular complex binding | 0.01235212 | 7 |
| MF | GO:0042802 | Identical protein binding | 0.021546755 | 12 |
| MF | GO:0042803 | Protein homodimerization activity | 0.021546755 | 8 |
| MF | GO:0045296 | Cadherin binding | 0.021546755 | 6 |
| MF | GO:0019899 | Enzyme binding | 0.024765826 | 6 |
| MF | GO:0008134 | Transcription factor binding | 0.024765826 | 5 |
| MF | GO:0004197 | Cysteine-type endopeptidase activity | 0.024765826 | 4 |
| MF | GO:0019900 | Kinase binding | 0.024765826 | 4 |

**Supplementary Table 3.** KEGG enrichiment analysis of 43 differentially expressed autophagy-related genes in DN

| **Category** | **ID** | **Description** | **Adj.P-value** | **Count** |
| --- | --- | --- | --- | --- |
| KEGG_pathway | hsa04140 | Autophagy - animal | 2.38486E-05 | 9 |
| KEGG_pathway | hsa05164 | Influenza A | 5.2716E-05 | 9 |
| KEGG_pathway | hsa04621 | NOD-like receptor signaling pathway | 6.13884E-05 | 9 |
| KEGG_pathway | hsa05222 | Small cell lung cancer | 0.00011716 | 7 |
| KEGG_pathway | hsa05417 | Lipid and atherosclerosis | 0.000118895 | 9 |
| KEGG_pathway | hsa05163 | Human cytomegalovirus infection | 0.000138996 | 9 |
| KEGG_pathway | hsa05131 | Shigellosis | 0.000237324 | 9 |
| KEGG_pathway | hsa05200 | Pathways in cancer | 0.000246091 | 12 |
| KEGG_pathway | hsa04068 | FoxO signaling pathway | 0.000403171 | 7 |
| KEGG_pathway | hsa05160 | Hepatitis C | 0.000984888 | 7 |
| KEGG_pathway | hsa04217 | Necroptosis | 0.000984888 | 7 |
| KEGG_pathway | hsa05130 | Pathogenic Escherichia coli infection | 0.002947916 | 7 |
| KEGG_pathway | hsa05169 | Epstein-Barr virus infection | 0.003118719 | 7 |
| KEGG_pathway | hsa04210 | Apoptosis | 0.003839771 | 6 |
| KEGG_pathway | hsa04136 | Autophagy - other | 0.004302888 | 4 |
| KEGG_pathway | hsa05165 | Human papillomavirus infection | 0.005805051 | 8 |
| KEGG_pathway | hsa05161 | Hepatitis B | 0.006899433 | 7 |
| KEGG_pathway | hsa05132 | Salmonella infection | 0.006899433 | 6 |
| KEGG_pathway | hsa04657 | IL-17 signaling pathway | 0.006968194 | 5 |
| KEGG_pathway | hsa05142 | Chagas disease | 0.008996022 | 5 |
| KEGG_pathway | hsa04668 | TNF signaling pathway | 0.012134794 | 5 |
| KEGG_pathway | hsa05167 | Kaposi sarcoma-associated herpesvirus infection | 0.012270679 | 6 |
| KEGG_pathway | hsa05170 | Human immunodeficiency virus 1 infection | 0.017349234 | 6 |
| KEGG_pathway | hsa05135 | Yersinia infection | 0.022198566 | 5 |
| KEGG_pathway | hsa05162 | Measles | 0.022455822 | 5 |
| KEGG_pathway | hsa04137 | Mitophagy - animal | 0.02644306 | 4 |
